# Residues in Human Arsenic (+3 Oxidation State) Methyltransferase Forming Potential Hydrogen Bond Network around S-adenosylmethionine

**DOI:** 10.1371/journal.pone.0076709

**Published:** 2013-10-04

**Authors:** Xiangli Li, Jing Cao, Shuping Wang, Zhirong Geng, Xiaoli Song, Xin Hu, Zhilin Wang

**Affiliations:** 1 State Key Laboratory of Coordination Chemistry, School of Chemistry and Chemical Engineering, Nanjing University, Nanjing, PR China; 2 School of Chemistry and Chemical Engineering, Yangzhou University, Yangzhou, PR China; 3 Modern Analysis Center of Nanjing University, Nanjing, PR China; Universität Stuttgart, Germany

## Abstract

Residues Tyr59, Gly78, Ser79, Met103, Gln107, Ile136 and Glu137 in human arsenic (+3 oxidation state) methyltransferase (hAS3MT) were deduced to form a potential hydrogen bond network around S-adenosylmethionine (SAM) from the sequence alignment between Cyanidioschyzon merolae arsenite S-adenosylmethyltransferase (CmArsM) and hAS3MT. Herein, seven mutants Y59A, G78A, S79A, M103A, Q107A, I136A and E137A were obtained. Their catalytic activities and conformations were characterized and models were built. Y59A and G78A were completely inactive. Only 7.0%, 10.6% and 13.8% inorganic arsenic (iAs) was transformed to monomethylated arsenicals (MMA) when M103A, Q107A and I136A were used as the enzyme. The *V_max_* (the maximal velocity of the reaction) values of M103A, Q107A, I136A and E137A were decreased to 8%, 22%, 15% and 50% of that of WT-hAS3MT, respectively. The *K_M(SAM)_* (the Michaelis constant for SAM) values of mutants M103A, I136A and E137A were 15.7, 8.9 and 5.1 fold higher than that of WT-hAS3MT, respectively, indicating that their affinities for SAM were weakened. The altered microenvironment of SAM and the reduced capacity of binding arsenic deduced from *K_M(As)_* (the Michaelis constant for iAs) value probably synergetically reduced the catalytic activity of Q107A. The catalytic activity of S79A was higher than that of WT despite of the higher *K_M(SAM)_*, suggesting that Ser79 did not impact the catalytic activity of hAS3MT. In short, residues Tyr59 and Gly78 significantly influenced the catalytic activity of hAS3MT as well as Met103, Ile136 and Glu137 because they were closely associated with SAM-binding, while residue Gln107 did not affect SAM-binding regardless of affecting the catalytic activity of hAS3MT. Modeling and our experimental results suggest that the adenine ring of SAM is sandwiched between Ile136 and Met103, the amide group of SAM is hydrogen bonded to Gly78 in hAS3MT and SAM is bonded to Tyr59 with van der Waals, cation-π and hydrogen bonding contacts.

## Introduction

S-adenosylmethionine (SAM), which is a conjugate of nucleotide adenosine and amino acid methionine, is an essential metabolic intermediate in every organism having several SAM-dependent enzymes [1]-[4]. One of the important biological roles of SAM as a cofactor is the transfer of its methyl group to different substrates, such as lipids, proteins, DNA and other small molecules (inorganic arsenic, chloride, bromide) [5]–[8]. All these methylation processes catalyzed by SAM-dependent methyltransferases significantly affect various essential biological processes including biosynthesis, protein repair, signal transduction, metabolism and detoxification [9], [10]. The methylation of arsenic catalyzed by arsenic (+3 oxidation state) methyltransferase (AS3MT) is considered to be the major metabolism pathway of arsenic [11], [12]. Besides AS3MT, the arsenic methylation process needs tris(2-carboxyethyl)-phosphine hydrochloride (TCEP), glutathione (GSH) or coupled reducing systems as the reductant and SAM as the methyl donor [12]–[16]. Mechanisms of arsenic methylation have been studied for many years [16], [17], but those of the methyl transfer from SAM to As atom have not been studied. Discovery of SAM and As-binding sites is a prerequisite for understanding the methylation of arsenic via hAS3MT.

Active and As-binding sites in AS3MT have been studied in earlier studies. Residues Cys157 and Cys207 in recombinant mouse AS3MT, Cys156 in rat AS3MT, Cys156 and Cys206 in human AS3MT (hAS3MT) and Cys72, Cys174 and Cys224 in Cyanidioschyzon merolae arsenite S-adenosylmethyltransferase (CmArsM) have been verified to be the As-binding sites and in the active sites of mouse, rat, human and cyanidioschyzon merolae AS3MT, respectively [18]–[21]. However, most of the SAM-binding sites in hAS3MT have been deduced from the sequence alignment between diverse species AS3MT [2], [11], [22], [23]. Only the function of the acidic residues of hAS3MT in the predicted SAM-binding motifs has been studied [24].

The residues in CmArsM, which form a potential hydrogen bond network around SAM deduced from the crystal structure of CmArsM-SAM, are Y70, G91, C92, D115, M116, Q120, I151, E152 and C174 [25]. The corresponding residues in hAS3MT are Y59, G78, S79, D102, M103, Q107, I136, E137 and C156. Gly78 and Ser79 belong to the SAM-binding motif I 74-ILDLGSGSG-82, which is inferred from the sequence alignment of various SAM-dependent methyltransferases [11], [23]. The functions of C156 and D102 have been studied [20], [24]. However, the functions of other residues have not been demonstrated. In order to determine whether residues Y59, G78, S79, M103, Q107, I136 and E137 bind to SAM, herein we designed seven mutants Y59A, G78A, S79A, M103A, Q107A, I136A and E137A by site-directed mutagenesis. The catalytic activities of the mutants were determined by high performance liquid chromatography-inductively coupled plasma-mass spectrometry (HPLC-ICP-MS), the mutant conformations were characterized by circular dichroism (CD) and attenuated total reflection Fourier transform infrared spectrometry (ATR-FTIR), and the models of hAS3MT-SAM were built by modeller9v8 utilizing the most updated protein template CmArsM with a sequence most similar to that of hAS3MT. We conclude that Tyr59 and Gly78 markedly influence the SAM-binding and catalytic activity of hAS3MT as well as Met103, Gln107, Ile136 and Glu137. The capacities of binding to SAM for mutants M103A, I136A and E137A are weaker than that of WT. Ser79 exerts no effect on the catalytic activity of hAS3MT. The *K_M_* value of SAM (*K_M(SAM)_*) for S79A suggests that Ser79 also affects the SAM binding slightly. Modeling of WT-hAS3MT with SAM shows that the adenine ring of SAM is sandwiched between Ile136 on one side and Met103 on the other side, the hydrogen bond is formed between the amide group of SAM and Gly78 rather than Ser79 estimated from the activity of the G78A and S79A. Tyr59 plays important roles in distinguishing and binding SAM, it might bond to the S^+^-CH_3_ of SAM with van der Waals contacts, the electron-rich π system of Tyr59 forms cation-π interaction with sulfonium S^+^-CH_3_ of SAM as well as the hydrogen bond formed between Tyr59 and the carboxyl group of SAM.

## Materials and Methods

*Caution*: Arsenical compounds are human carcinogens and should be handled accordingly [26].

### Materials

Expression host, *Escherichia coli* BL21 (DE3) pLysS, was bought from Novagen. Restriction enzymes, dNTPs and PrimerSTAR HS DNA polymerase, were obtained from Takara. Wild-type hAS3MT expression plasmid, pET-32a-hAS3MT, was derived from an earlier study [27]. SAM, GSH, isopropyl β-D-thiogalactopyranoside (IPTG) and bovine serum albumin (BSA) were purchased from Sigma. Arsenicals were bought from J&K Chemical Ltd. Phosphate-buffered saline (PBS, pH 7.0) buffer was prepared by mixing appropriate volumes of Na_2_HPO_4_ and NaH_2_PO_4_ into a 25 mM stock solution.

Stock solutions, which contained 25000 µg/L of the following arsenic species each, were prepared using Milli-Q deionized water with NaAsO_2_ (As^3+^), Na_2_HAsO_4_.7H_2_O (As^5+^), disodium methylarsonate (MMA^5+^) and dimethylarsonate acid (DMA^5+^) (J&K Chemical Ltd.). All of the four stock solutions were stored at 4 °C in the dark, by which the working solutions of standards were prepared fresh daily.

### Preparation of hAS3MT mutants

Tyr59, Gly78, Ser79, Met103, Gln107, Ile136 and Glu137 were mutated to Ala by site-directed mutagenesis using the wild type pET-32a-*hAS3MT* plasmid as a template [20], [27]. The primers used for site-directed mutagenesis were summarized in Table 1. The PCR product was transformed into *E. coli* Top10 (Invitrogen) cells. After sequencing [28], the vectors carrying mutant hAS3MT genes were transformed into *E. coli* BL21 (DE3) pLysS for expression and a single colony was selected from standard ampicillin-containing agar plate. Protein expression and purification were performed according to the protocols described previously [27]. The method of Bradford based on a BSA standard curve was used to determine protein concentration [29].

**Table 1:** Primers used for site-directed mutagenesis.

	Primer Sequence
Y59A	+ 5'- GTAGCCCTAAGATAT**GCG**GGCTG-3'
	– 5'- CAGACCACAGCC**CGC**ATATCTTAGG-3'
G78A	+ 5’ -TGCTGGATTTTGGATCTG**GCG**AGTGG-3’
	– 5’-TCTACCACTTCCACT**CGC**CAGATCC-3’
S79A	+ 5'–GATTTTGGATCTGGGT**GCG**GGAAG -3'
	– 5'–AATCTCTACCACTTCC**CGC**ACCC -3'
M103A	+ 5' –GACACGTGACTGGAATAGAC**GCG**AC-3'
	– 5'- TGGCCTTTGGT**CGC**GTCTATTCC-3'
Q107A	+ 5'- ATAGACATGACCAAAGGC**GCG**GTG -3'
	– 5'- TTTCAGCCACTTCCAC**CGC**GCCTTTG-3'
I136A	+ 5'- GACTTTTATTCATGGCTAC**GCG**GAG-3'
	– 5'- CTCTCCCAACTTCTC**CGC**GTAG-3'
E137A	+ 5'- ATGGCTACATT**GCG**AAGTTGGGAG -3'
	– 5'- CTCTCCCAACTT**CGC**AATGTAGCC -3'
Whole	+ 5'-CGGGATATCATGGCTGCACTTCGTGAC-3'
	– 5'-CGGGTCGACTTAGTGATGGTGATG-3'

### Enzyme activity assays

The steady-state activity of the mutants was determined with an assay system (100 µl) containing 11 µg enzyme, 7 mM GSH, 1 µM iAs^3+^ and 1 mM SAM in PBS (25 mM, pH 7.0) by HPLC-ICP-MS [20]. To measure the iAs^3+^ substrate kinetics, 1 mM SAM and 0.5-500 µM iAs^3+^ were used. In SAM kinetic experiments, 1 µM iAs^3+^ and 0.05-1 mM SAM were used. The reaction mixtures were incubated at 37 °C for 2 h, and then terminated by adding H_2_O_2_ to a final concentration of 3% to release the arsenicals from proteins and to oxidize all arsenic metabolites to pentavalency [16], [30]. 20 µl aliquots of the samples were separated on an anion-exchange column (PRP X-100 250 mm×4.6 mm i.d., 5 µm, Hamilton) by HPLC and analyzed by ICP-MS (Elan 9000, PerkinElmer) with the flow rate of 1.0 ml/min at room temperature [31]-[33]. The arsenical compounds were eluted with the mobile phase of 12 mM (NH_4_)_2_HPO_4_, the pH of which was adjusted to 6.0 with H_3_PO_4_. The amounts of arsenic species were calculated with the working curves prepared using 5, 10, 25, 50 and 100 µg/L of standard arsenic species. The methylation rates were calculated as mole equivalents of methyl groups that were transferred from SAM to iAs^3+^ (i.e., 1 nmol CH_3_ per 1 nmol MMA or 2 nmol CH_3_ per 1 nmol DMA) [34]. The methylation rate follows the noncompetitive substrate inhibition equation (1): *V = *[S]*V_max_/(K_M_+[S]*+*[S]^2^ */K_I_)* and double reciprocal equation (2): 1/*V = K_M_*/(V_max_*[S]) + 1/*V_max_* [20], [35], where *V* is the initial velocity of the reaction (pmol CH_3_ transferred/h/mg protein); [S], the substrate (iAs^3+^) concentration (µM); *V_max_*, the maximal velocity of the reaction (pmol CH_3_ transferred/h/mg protein); *K_M_*, the Michaelis constant for iAs^3+^ or SAM (µM); *K_I_*, the inhibition constant for iAs^3+^ (µM) [36].

### CD and ATR-FTIR spectra

CD spectra of the WT and hAS3MT mutants were recorded on a JASCO-J810 spectropolarimeter (Jasco Co., Japan) in a 1 mm cell and 10 mm light length. The scanning rate was set at 50 nm/min. Each spectrum represents the average of three accumulations recorded between 190- and 265-nm with very dilute enzyme solution of every mutant (2 µM in 25 mM PBS, pH 7.0) at room temperature. Baseline correction was automatically carried out with the PBS (25 mM, pH 7.0) spectrum throughout the entire collection. The secondary structure parameters of the mutants were calculated using Jwsse32 software with reference CD-Yang. jwr [37]. ATR-FTIR spectra were also utilized to analyze the secondary structure of the mutants. More details about the ATR-FTIR spectra were detailed in previous literature [20], [38]–[39].

### Modeling of hAS3MT mutants using modeller9v8

Models of hAS3MT-SAM were built by modeller9v8 utilizing the most updated protein template CmArsM (PDB code 4FR0), because both hAS3MT and CmArsM were arsenic methyltransferases in different species with similar sequences [25]. The models quality of hAS3MT mutants was estimated via QMEAN Server (http://swissmodel.expasy.org/qmean/cgi/index.cgi) [40]. Pymol was used to analyze the models of hAS3MT [41].

## Results and Discussion

### Expression and purification of the hAS3MT mutants

**Figure 1:**
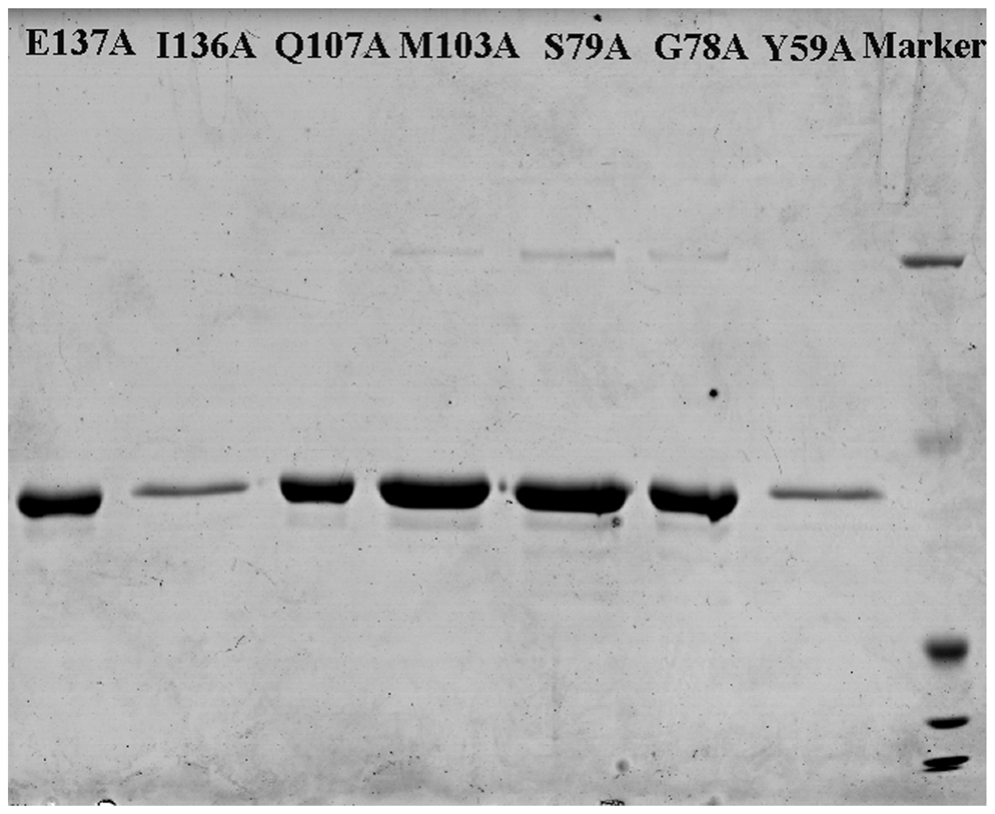
Sodium dodecyl sulfate polyacrylamide gel electrophoresis (SDS-PAGE). Coomassie blue stained 12% SDS-PAGE gel of the purified protein of hAS3MT mutants.

The hAS3MT mutants were expressed and purified according to the protocol described in previous studies [20], [27]. All the mutant proteins were expressed successfully. The purity of each mutant protein was confirmed to be over 90% by sodium dodecyl sulfate polyacrylamide gel electrophoresis (SDS-PAGE) (Figure 1).

### Catalytic activities of the mutants

**Figure 2:**
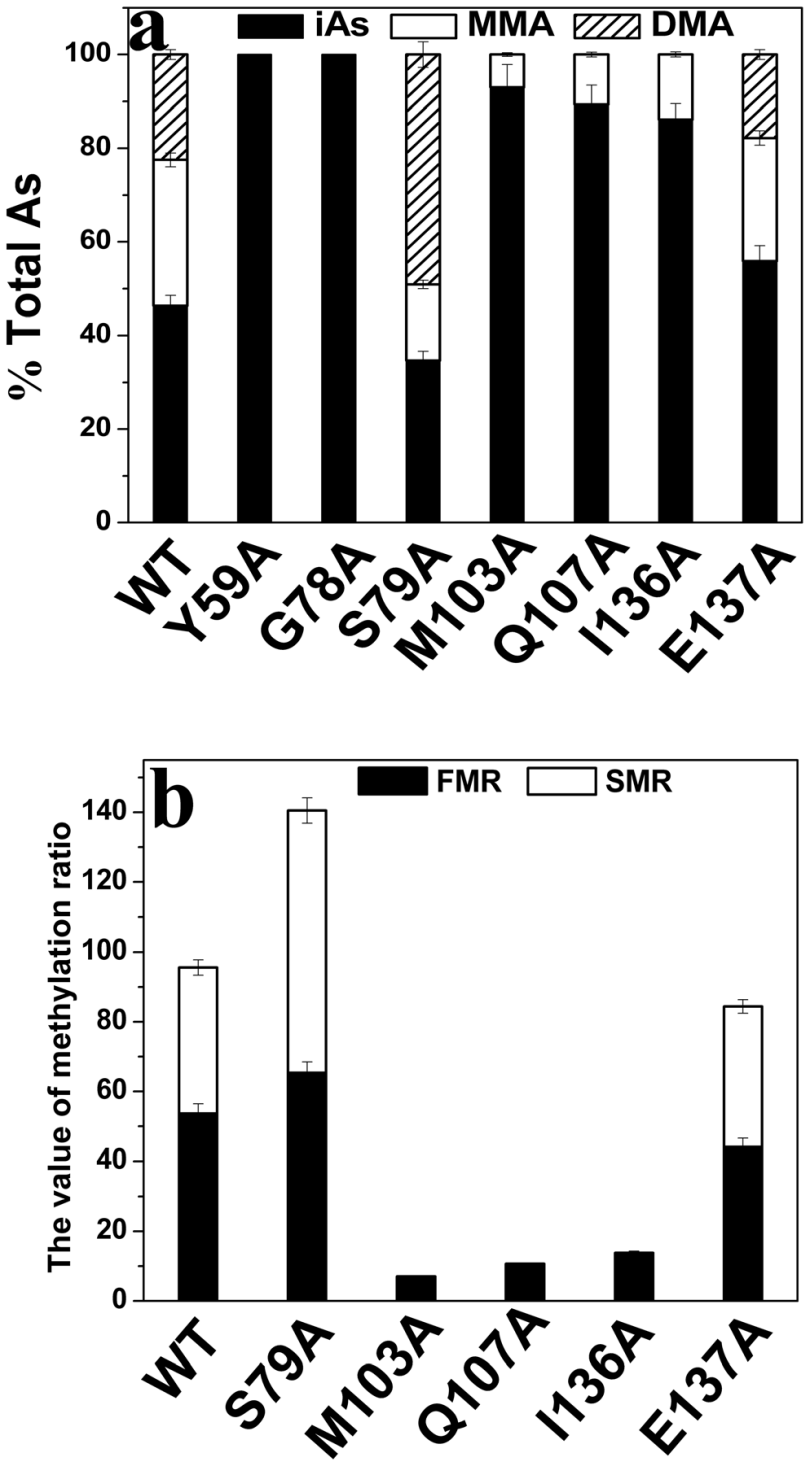
Catalytic capacity of the hAS3MT mutants. Reaction mixtures (100 µl) containing 11 µg enzymes, 1 µM iAs^3+^, 1 mM SAM and 7 mM GSH in PBS (25 mM, pH 7.0) were incubated at 37°C for 2 h with H_2_O_2_ treatment before analyzed by HPLC-ICP-MS. The percents of arsenic species (iAs/TAs, MMA/TAs and DMA/TAs) and the two indices (FMR and SMR) of mutants Y58A, G78A, S79A, M103A, Q107A, I136A and E137A are shown in Figure 2a and 2b. Values are the averages ±S.D. of three independent experiments performed by three independently purified proteins.

The primary methylation index (PMI) calculated as MMA/iAs and the secondary methylation index (SMI) calculated as DMA/MMA were previously developed to characterize iAs methylation ability [42], [43]. The total arsenic (TAs) concentration was defined by summing up the concentrations of iAs, MMA and DMA because no trimethylarsine oxide (TMAO) was detected in our reaction system. According to the pathway of iAs methylation, secondary methylation can only proceed based on first methylation and parts of the first methylation products are further methylated. Therefore, to assess the first methylation ability, not only the primary but also the secondary methylation products were considered. The first methylation ratio (FMR) was defined as (MMA + DMA)/TAs and the secondary methylation ratio (SMR) was defined as DMA/(MMA + DMA) [44]. Using the FMR and SMR to evaluate the arsenic methylation capacity of the mutants was more logical than using the PMI and SMI [45]. Proportions of iAs, MMA and DMA (iAs%, MMA% and DMA%) were defined as iAs/TAs × 100%, MMA/TAs × 100% and DMA/TAs × 100%, respectively [44]. Proportions of arsenic species and the two methylation indices, FMR and SMR were calculated to evaluate the arsenic methylation capacity of the mutants Y59A, G78A, S79A, M103A, Q107A, I136A and E137A (Figure 2a and Figure 2b). Mutants Y59A and G78A were completely inactive owing to the absence of methylated arsenic. The proportion of iAs of S79A was lower than that of WT while the FMR and SMR of S79A were higher than that of WT, which indicated that the methylation capacity of S79A was stronger than that of WT. Only 7.0%, 10.6%, 13.8% iAs was transformed to MMA when M103A, Q107A and I136A were used as the enzyme, which suggested that the catalytic activities of mutants M103A, Q107A, I136A and E137A were all lower than that of WT. Compared with WT, the catalytic capacity of E137A also decreased as concluded from the proportion of iAs, FMR and SMR. Thus, residues Tyr59 and Gly78 affected the activity of hAS3MT significantly whereas Met103, Gln107, Ile136 and Glu137 influenced the catalytic activity to a lesser extent, and Ser79 had no effect.

**Figure 3:**
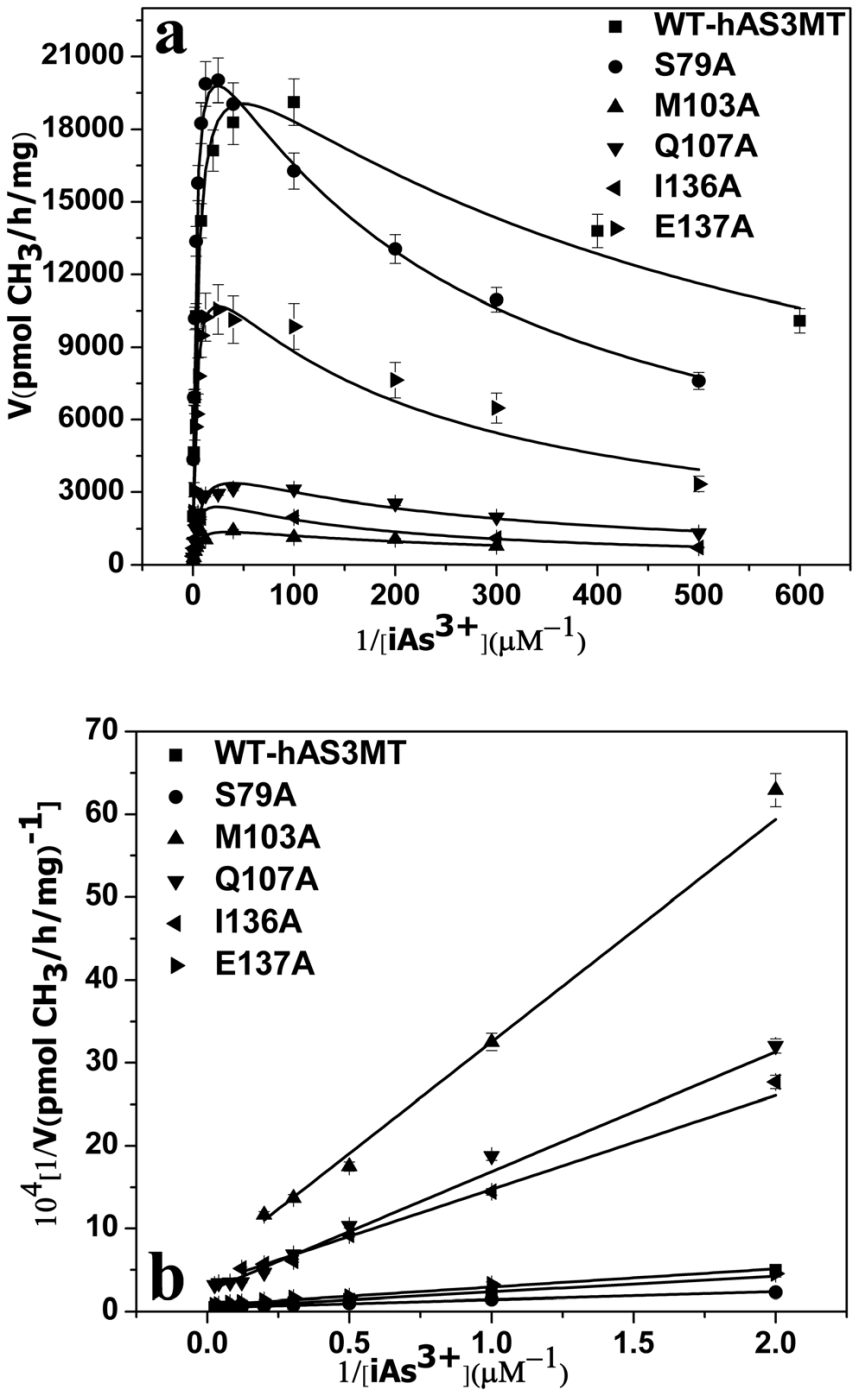
A: Substrate concentration dependence of rate. The lines show the least squares fit of Eq. (1) to the data. **B: Double reciprocal plots of the arsenic methylation rate against the concentration of iAs^3+^.** Reaction mixtures (100 µl) containing 11 µg enzymes, 1 mM SAM and 7 mM GSH in PBS (25 mM, pH 7.0) were incubated with different concentrations of iAs^3+^ at 37°C for 2 h with H_2_O_2_ treatment before analysis. Values are the averages ± S.D. of three independent experiments performed by three independently purified proteins.

The catalytic activities and kinetic properties of the mutants were investigated comprehensively. The iAs^3+^-substrate-inhibition phenomena were observed for all the active mutants in a wide iAs^3+^ concentration range (0.5–500 µM) (Figure 3a), namely the rate of arsenic methylation increased with increasing arsenic concentration (0.5–40 µM) and then decreased at higher arsenic concentration (100–500 µM). The kinetic parameters of the active mutants in Table 2 were estimated by fitting the experimental data to Eq. (1) and calculated by double reciprocal plots from Eq. (2) (Figure 3b). The data obtained from the two methods were consistent. The *K_I_* values for the iAs^3+^ of mutants S79A, M103A, Q107A, I136A and E137A were all lower than those of WT-hAS3MT (*K_M_*, 3.2 µM, *K_I_*, 0.7 mM, *V_max_*, 19836 pmol/h/mg [20]). The *V_max_* values of M103A, Q107A, I136A and E137A were decreased to 8%, 22%, 15% and 50% of that of WT-hAs3MT respectively, while the *V_max_* value of S79A was higher than that of WT. The *K_M(As)_* values of M103A and Q107A were higher than that of WT. For S79A, I136A and E137A, the *K_M(As)_* values did not obviously differ from that of WT-hAS3MT. The results indicated that the affinity of mutants M103A and Q107A to iAs decreased compared with that of WT, while the abilities in binding the substrate iAs^3+^ of mutants S79A, I136A and E137A were similar to the native form.

**Table 2:** Kinetic parameters of arsenic methylation for the five mutants S79A, M103A, Q107A, I136A and E137A.

	aV_max_(pmol CH_3_ /mg/h)×10^3^	aK_M_(µM)	K_I_(mM)	bV_max_(pmol CH_3_ /mg/h) ×10^3^	^B^K_M_ (µM)	^C^K_M_ (µM)	Relative cK_M_
S79A	23.7±0.5	2.4±0.2	0.24±0.01	22.3±0.5	2.1±0.1	93.1±2.4	1.9
M103A	1.7±0.2	4.6±0.8	0.27±0.08	1.8±0.2	4.8±0.1	750.4±25.6	15.7
Q107A	4.6±0.3	7.0±0.8	0.22±0.03	4.1±0.4	6.0±0.2	50.2±2.0	1.0
I136A	3.1±0.3	3.7±0.5	0.16±0.02	3.0±0.3	3.4±0.5	426.7±17.9	8.9
E137A	13.2±1.0	3.0±0.5	0.21±0.04	13.5±0.8	3.0±0.2	241.9±23.5	5.1
WT	21.2±1.1	3.2±0.3	0.76±0.09	19.8±1.0	3.19±0.7	47.8	1.0

Values represent the average ± S.D. of three independent experiments performed by three independently purified proteins.

a Represents the kinetic parameters of iAs^3+^ estimated from the data in Fig. 2a by Eq. (1) using origin 8.0.

b Represents the kinetic parameters of iAs^3+^ calculated from the data in Fig. 2b.

c Represents the *K_M_* for SAM.

**Figure 4:**
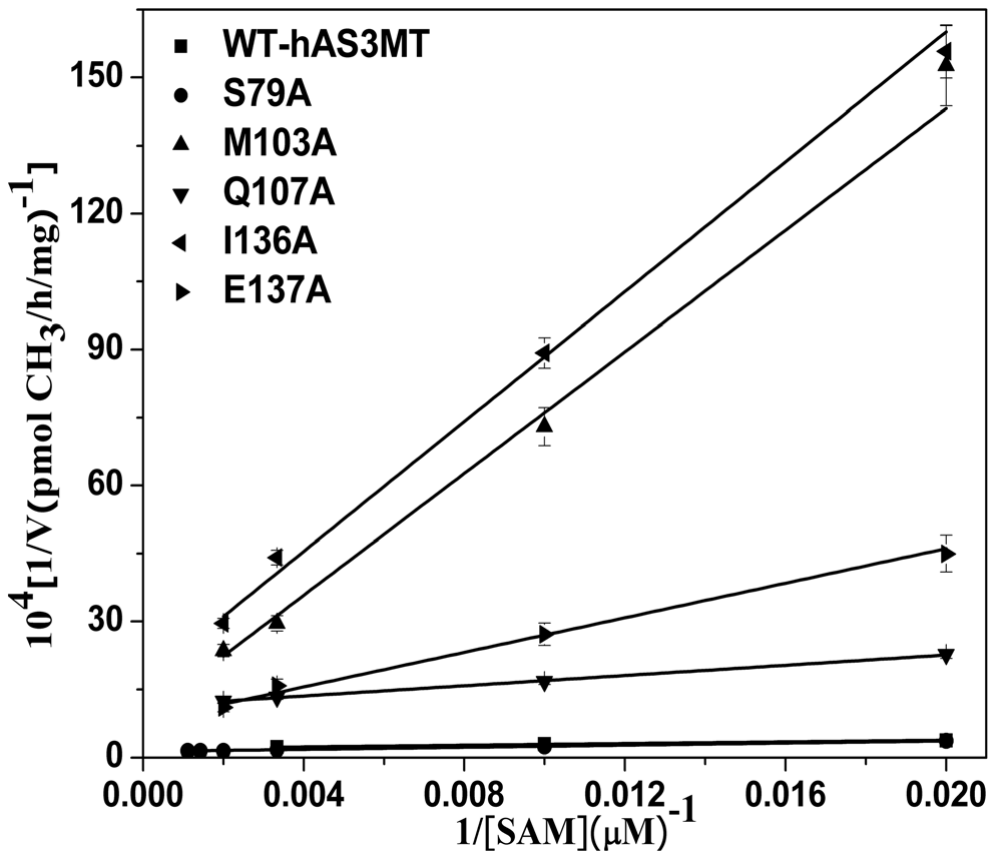
Double reciprocal plots of the arsenic methylation rate versus the concentration of SAM. Reaction mixtures (100 µl) containing 11 µg enzymes, 1 µM iAs^3+^, 7 mM GSH in PBS (25 mM, pH 7.0) were incubated with different concentrations of SAM for 2 h with H_2_O_2_ treatment before analysis. Values are the averages ± S.D. of three independent experiments performed by three independently purified proteins.

The arsenic methylation rate increased with rising SAM concentration (Figure 4). The *K_M_*_(SAM)_ values of the mutants, which reflect the ability of SAM to interact with hAS3MT, were calculated from double reciprocal plots (Figure 4) and summarized in Table 2. For the mutants M103A, I136A and E137A, the *K_M_*_(SAM)_ values increased to 750.4, 426.7 and 241.9 µM, which were 15.7, 8.9 and 5.1 fold higher than the WT value (WT: 47.84 µM [20]), respectively. The data revealed that residues Met103, Ile136 and Glu137 were involved in the cofactor SAM binding, which is reflect by the reduced catalytic activity of M103A, I136A and E137A. The *K_M_*_(SAM)_ of S79A was about 2 fold higher than that of WT, while the *K_M_*_(SAM)_ of Q107A almost equaled that of WT though the catalytic activity of Q107A was evidently lower.

### Conformation of Y59A, G78A, S79A, M103A, Q107A, I136A and E137A

**Figure 5:**
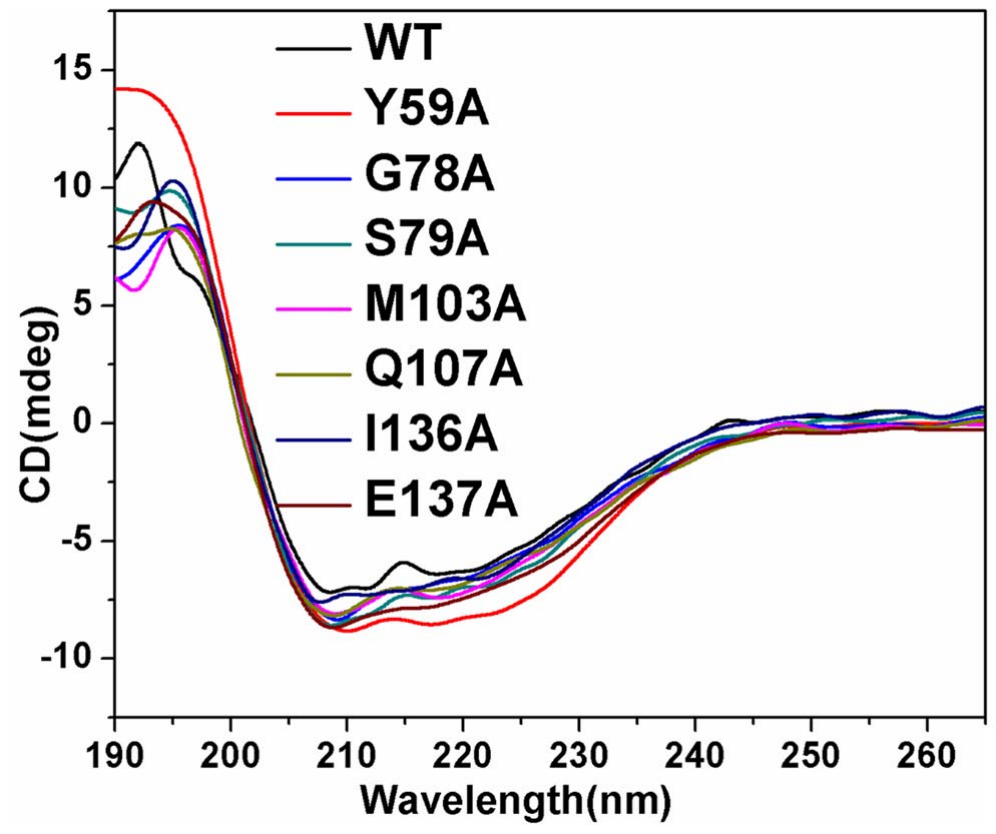
CD spectra of hAS3MT and the mutants. Spectra were taken at the protein concentration of 2 µM at room temperature. Plot is the representative of three independent measurements performed by three independently purified proteins.

Circular dichroism (CD) spectroscopy, which is a sensitive method to determine the protein secondary structure [46], [47], is used to analyze the conformational change of the seven mutants (Figure 5). CD spectra showed that the intensities of the peaks (208 and 220 nm) of mutants Y59A, G78A, S79A, M103A, Q107A, I136A and E137A were more intense than those of the wild type enzyme suggesting that the conformations of mutants Y59A, G78A, S79A, M103A, Q107A, I136A and E137A were different from that of WT. The content of secondary structure of the mutants was computed by Jwsse32 software with reference CD-Yang. jwr (Table 3). Compared with WT, the contents of β-pleated sheet for all mutants except Y59A increased with those of β-turn and random coil decreasing, while the content of α-helix of Y59A increased with those of β-pleated sheet, β-turn and random coil decreasing. The data indicate that the contents of secondary structure of the seven mutants differ from those of WT, especially for Y59A and I136A.

**Table 3:** Secondary structures of WT-hAS3MT and the mutants estimated from CD spectra.

	α-helix%	β-pleated%	β-turn%	Random%
Y59A	41.3±1.9	17.6±0.5	13.4±0.5	27.7±1.6
G78A	27.0±1.1	32.3±1.1	15.1±0.9	25.5±0.2
S79A	29.7±1.6	30.7±1.3	13.3±0.8	26.3±0.5
M103A	26.6±0.3	30.3±2.0	16.2±0.9	26.9±0.8
Q107A	27.2±1.2	31.2±0.8	13.1±1.0	28.4±0.3
I136A	25.0±1.0	44.0±2.9	7.8±1.2	23.2±0.7
E137A	30.0±1.2	25.6±1.1	16.3±0.7	28.1±1.2
WT	29.0±2.2	23.9±1.9	17.9±1.7	29.2±1.4

Values represent the average ± S.D. of three independent experiments carried out by three independently purified proteins. The parameters were analyzed with the Jasco secondary structure manager with the reference CD data-Yang. jwr in PBS (25 mM, pH 7.0) at room temperature.

**Figure 6:**
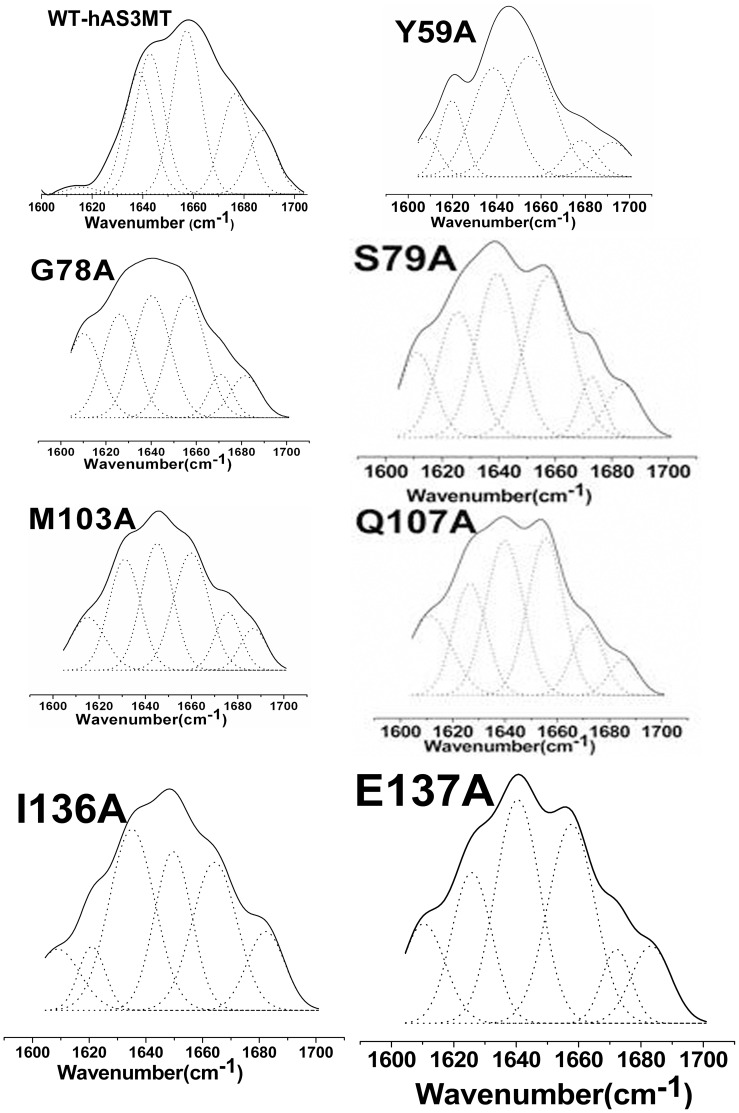
Curve-fitted amide I region of the mutants. The component peaks are the result of curve-fitting using a Gaussian shape. The solid lines represent the experimental FTIR spectra after Savitzky-Golay smoothing, and the dashed lines represent the fitted components. Plot is the representative of three independent measurements carried out by three independently purified proteins.

FTIR spectroscopy has also been shown to be a powerful technique to evaluate the protein structure. To further confirm the secondary structure of the mutants, we carried out ATR-FTIR assays and analyzed their amide I band spectra according to well-established assignment criteria [38], [48] (1610–1640 cm^−1^: β-pleated sheet, 1640–1650 cm^−1^: random coil, 1650–1658 cm^−1^: α-helix, and 1660–1700 cm^−1^: β-turn). The original and curve-fitting FTIR spectra of mutants Y59A, G78A, S79A, M103A, Q107A, I136A and E137A were shown in Figure 6. There were six component bands in the amide I bands of the mutants. The contents of each secondary structure were calculated from the integrated areas of the component bands (Table 4). The secondary structure derived from ATR-FTIR is in good agreement with those from CD spectra.

**Table 4:** Secondary structures of WT-hAS3MT and the mutants estimated from ATR-FTIR spectroscopy.

	α-helix%	β-pleated%	β-turn%	Random%
Y59A	38.9±3.1	17.0±0.8	14.2±0.8	29.9±2.0
G78A	26.4±0.5	33.8±0.3	13.4±0.3	26.3±0.8
S79A	29.9±1.0	29.9±0.4	13.1±0.3	27.1±0.3
M103A	26.0±1.7	32.7±2.5	16.3±1.7	24.9±1.1
Q107A	26.7±0.8	30.6±1.3	15.3±0.3	27.4±0.5
I136A	24.1±1.8	43.6±1.7	10.8±0.9	21.5±0.6
E137A	27.3±2.3	27.8±2.1	15.5±1.1	29.4±1.6
WT	26.6±3.6	20.7±4.6	24.2±3.2	28.5±4.9

### SAM-binding sites of hAS3MT mutants Y59A, G78A, S79A, M103A, Q107A, I136A and E137A

**Figure 7:**
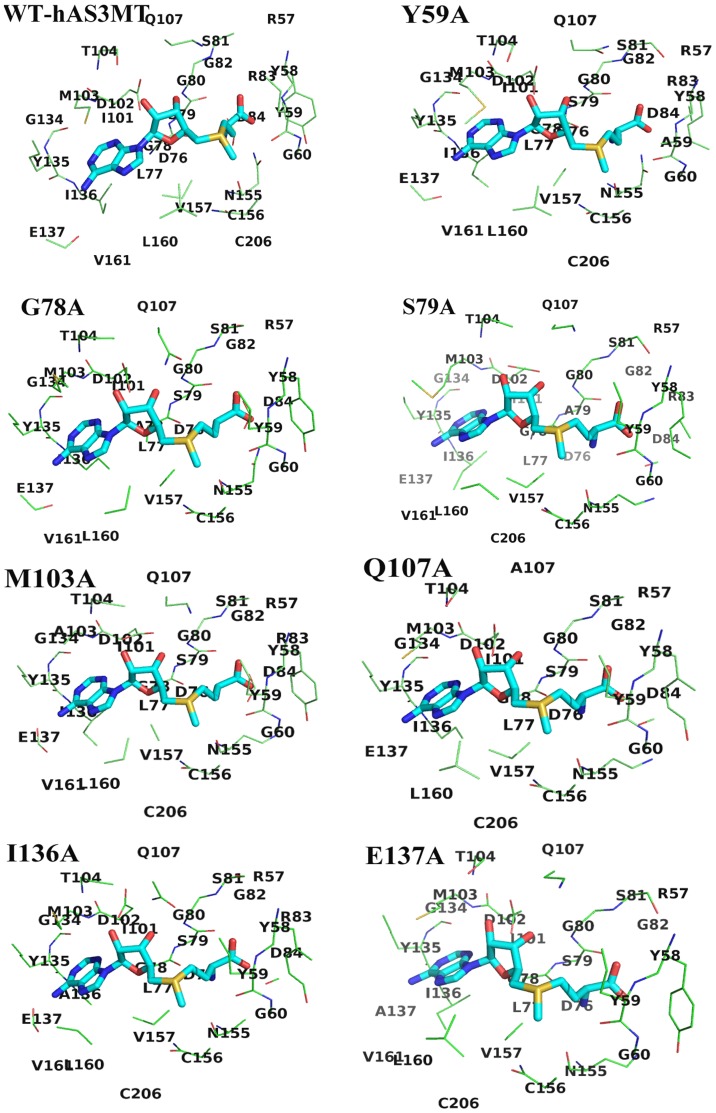
Interaction modes between SAM, WT-hAS3MT and mutants (Y59A, G78A, S79A, M103A, Q107A, I136A and E137A). Only the residues 5.0 Å around SAM are displayed.

The models of hAS3MT mutants were established using modeller9v8. The crystal structure of CmArsM with cofactor SAM (PDB code 4FR0) was used as the template. The model quality was estimated on the basis of a QMEAN scoring function acceptably ranging between 0.60 and 0.65 [40]. The secondary structure arrangement of the hAS3MT model was almost identical to that of CmArsM. The sites in the SAM-binding pocket (5.0 Å around SAM) of WT-hAS3MT and mutants (Y59A, G78A, S79A, M103A, Q107A, I136A and E137A) were displayed in Figure 7 and Table 5. The SAM model-derived WT-hAS3MT showed that As-binding sites Cys156 and Cys206 were located in the SAM-binding pocket [24]. However, Cys206 was not in the SAM-binding pocket of mutant G78A, which induced complete catalytic activity loss by isolating As from S^+^-CH_3_, thus further demonstrating that G78A was completely inactive in the methylation of iAs. Another residue Arg83 was not located in the 5 Å range around SAM for G78A. The structure of Gly was simpler than that of Ala, so the microenvironment around SAM and the interaction between SAM and residue 78 changed when the Gly78 was replaced by Ala. Residues Arg83 and Val161 were not located in the 5 Å range around SAM for Q107A. In mutant E137A, the residues 5 Å around SAM were disconnected from R83 and D84. Gln and Glu are hydrophilic and their side chains are amide and carboxyl group, while Ala is hydrophobic and its side chain is smaller than that of Gln and Glu. The microenvironment around SAM changed and the catalytic activity of Q107A and E137A decreased when the residues 107 and 137 were changed to Ala. Residues around SAM for mutants Y59A, S79A, M103A and I136A were the same as that for WT.

**Table 5:** Residues 5.0 Å around SAM based on the models of WT and mutants Y59A, G78A, S79A, M103A, Q107A, I136A and E137A.

	Residues around 5.0 Å of SAM
WT	57-RYYG-60,76-DLGSGSGRD-84,101-IDMT-104,Q107,134-GYIE-137,155-NCV-157,160-LV-161,C206
Y59A	57-RYAG-60,76-DLGSGSGRD-84,101-IDMT-104,Q107,134-GYIE-137,155-NCV-157,160-LV-161,C206
G78A	57-RYYG-60,76-DLASGSG-82,D84,101-IDMT-104,Q107,134-GYIE-137,155-NCV-157,160-LV-161
S79A	57-RYYG-60,76-DLGAGSGRD-84,101-IDMT-104,Q107,134-GYIE-137,155-NCV-157,160-LV-161,C206
M103A	57-RYYG-60,76-DLGSGSGRD-84,101-IDAT-104,Q107,134-GYIE-137,155-NCV-157,160-LV-161,C206
Q107A	57-RYYG-60,76-DLGSGSG-82,D84,101-IDMT-104,A107,134-GYIE-137,155-NCV-157,L160,C206
I136A	57-RYYG-60,76-DLGSGSGRD-84,101-IDMT-104,Q107,134-GYAE-137,155-NCV-157,160-LV-161,C206
E137A	57-RYYG-60,76-DLGSGSG-82,101-IDMT-104,Q107,134-GYIA-137,155-NCV-157,160-LV-161,C206

One of the crucial interactions between the SAM having cation sulfonium R_3_S^+^ and SAM-dependent methyltransferases is the cation-π force formed between aromatic residues (e.g. Tyr, Trp and Phe) with their electron-rich π system and R_3_S^+^ of SAM [49]–[52]. The cation-π interaction formed between Tyr59 and S^+^-CH_3_ of SAM assists to bind, recognize SAM and distinguish cationic SAM from its analogous neutral molecule S-adenosylhomocysteine (SAH). The environment around the SAM-binding sites facilitates transfer of a methyl group from SAM to As atom. These interactions between SAM and residue 59 were destroyed when Tyr59 was replaced by Ala without electron-rich aromatic side chain, and therefore resulted in Y59A being an inactive enzyme.

**Figure 8:**
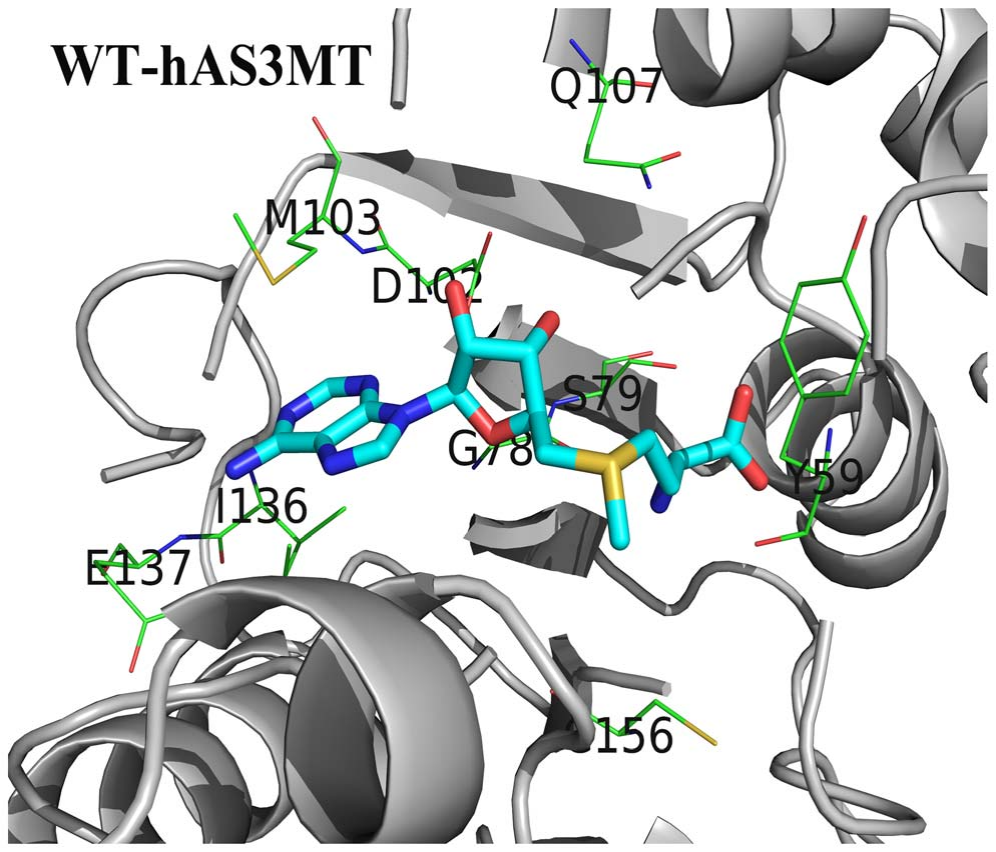
The model of WT-hAS3MT with SAM. Only residues in hAS3MT forming potential hydrogen bond network around SAM are presented.

SAM is bonded to CmArsM via forming hydrogen bonds and hydrophobic interactions with CmArsM residues [25]. The crystal structure of CmArsM-SAM showed that the adenine ring of SAM is sandwiched by Ile151 on one side and Met116 on the other side and forms hydrogen bonds with Ile151 and Glu152, the other hydrogen bonds also form between hydroxyl groups of ribose O2* and O3* and the side chains of Asp115 and Gln120, the carboxyl group of SAM and O^Cys92^ and O^Tyr70^, and the amide group of SAM and O^Gly91^ in CmArsM, the S^+^-CH_3_ group of SAM is bonded to main chain atoms O^Tyr70^ and O^Cys174^ in CmArsM with van der Waals contacts [25]. As detailed above, the residues Tyr70, Gly91, Cys92, Asp115, Met116, Gln120, Ile151, Glu152 and Cys174 in CmArsM correspond to the residues Tyr59, Gly78, Ser79, Asp102, Met103, Gln107, Ile136, Glu137 and Cys156 in hAS3MT. The results in this study and others show that the mutants Y59A, G78A, D102P, D102N and C156S are all completely inactive [20], [24], S79A is a little more active than WT while the catalytic activity of M103A, Q107A, I136A and E137A are decreased compared with that of WT. In our models, the adenine ring is indeed sandwiched between Ile136 on one side and Met103 on the other side because their aliphatic side chains are large enough to enwrap the adenine ring of SAM (Figure 8). The side chain of Ala was much smaller than that of Met103 and Ile136, the residue 103 and 136 would not enwrap the adenine ring of SAM when they were replaced by Ala. Asp102 forms two hydrogen bonds with the ribose hydroxyl groups of SAM [24]. The *V_max_* and *K_M(SAM)_* value of Q107A shows that Gln107 also significantly affects the catalytic activity of hAS3MT but it does not relate with the SAM-binding. Cys156 with functional group –SH, which is one of the As-binding sites in hAS3MT, also interacts with the S^+^-CH_3_ of SAM and helps to orient the methyl group of SAM during its approach to the arsenic lone pair. Both Gly78 and Ser79 belong to the SAM-binding motif I 74-ILDLGSGSG-82 and are close to the SAM in the models (Figure 8). The amide group of SAM is hydrogen bonded to Gly78 in hAS3MT rather than Ser79 as estimated from the activity of the G78A and S79A. According to the function of Tyr70 in CmArsM and the electron-rich characteristic of residue Tyr, we conclude that the O^Tyr59^ in hAS3MT might bond to the S^+^-CH_3_ of SAM with van der Waals contacts, the electron-rich π system of Tyr59 forms a cation-π interaction with sulfonium S^+^-CH_3_ of SAM as well as hydrogen bonding with the carboxyl group of SAM. Therefore, the Tyr59 plays an important role in binding, recognizing, distinguishing cationic SAM from its analogous neutral molecule SAH and orienting the methyl group of SAM during its approach to the arsenic electron lone pair.

## Conclusion

To study the functions of residues Tyr59, Gly78, Ser79, Met103, Glu107, Ile136 and Glu137 in hAS3MT, seven mutants, Y59A, G78A, S79A, M103A, Q107A, I136A and E137A were designed. Their conformation characterized by CD and ATR-FTIR spectroscopy was different from that of WT-hAS3MT. The *K_M(SAM)_* of mutants M103A and I136A indicated that they bound to SAM less tightly than WT-hAS3MT did, leading to remarkably lowered catalytic activity. The mutant Y59A was completely inactive because the cation-π interacton and hydrogen bond formed between SAM and residue 59 disappeared when Tyr59 was changed to Ala. The modeling results showed that As-binding sites Cys156 and Cys206 in WT-hAS3MT were located in the vicinity of SAM (5 Å around), whereas As-binding site Cys206 in mutant G78A was not, which resulted in the mutant G78A being inactive. The microenvironment surrounding SAM in Q107A was also altered, and its capacity of binding to iAs was weaker, which caused a dramatic decrease in the catalytic activity of the Q107A mutant. The catalytic activity of S79A surpassed that of WT although its *K_M(SAM)_* value was higher. Met103 and Ile136 form a sandwiched structure with the adenine ring of SAM in the center. The amide group of SAM is hydrogen bonded to O^Gly78^ in hAS3MT, while the hydrogen bond might not be formed between Ser79 and SAM as estimated from the activity of the G78A and S79A. O^Tyr59^ might bond to the S^+^-CH_3_ of SAM through van der Waals contacts, the electron-rich π system of Tyr59 could interact with sulfonium S^+^-CH_3_ of SAM, and the hydroxyl group might hydrogen bond with the carboxyl group of SAM. These interactions would help to bind, recognize, distinguish cationic SAM from its analogous neutral molecule SAH and orient the methyl group of SAM during its approach to the arsenic electron lone pair.
